# Self-weighing frequency and the incidence of type 2 diabetes: post hoc analysis of a cluster-randomized controlled trial

**DOI:** 10.1186/s13104-020-05215-x

**Published:** 2020-08-08

**Authors:** Naoki Sakane, Yoshitake Oshima, Kazuhiko Kotani, Akiko Suganuma, Shinsuke Nirengi, Kaoru Takahashi, Juichi Sato, Sadao Suzuki, Kazuo Izumi, Masayuki Kato, Mitsuhiko Noda, Hideshi Kuzuya

**Affiliations:** 1 Division of Preventive Medicine, Clinical Research Institute, National Hospital Organization Kyoto Medical Center, 1-1 Fukakusamukaihata-cho, Fushimi-ku, Kyoto, 612-8555 Japan; 2 Faculty of Humanities and Social Sciences, University of Marketing and Distribution Sciences, Hyogo, Japan; 3 Division of Community and Family Medicine, Jichi Medical University, Tochigi, Japan; 4 Hyogo Health Service Association, Hyogo, Japan; 5 Department of General Medicine/Family & Community Medicine, Nagoya University Graduate School of Medicine, Aichi, Japan; 6 Department of Public Health, Nagoya City University Graduate School of Medical Sciences, Aichi, Japan; 7 National Center for Global Health and Medicine, Tokyo, Japan; 8 Toranomon Hospital Health Management Center, Tokyo, Japan; 9 Ichikawa Hospital, International University of Health and Welfare, Chiba, Japan; 10 Koseikai Takeda Hospital, Kyoto, Japan

**Keywords:** Self-weighing, Pedometer, Diabetes prevention

## Abstract

**Objectives:**

Frequent self-weighing is associated with weight loss and maintenance, but the relationship between frequent self-weighing and the incidence of type 2 diabetes (T2D) remains unclear. The study aim was to examine the association between self-weighing frequency and the incidence of T2D in people with impaired fasting glucose (IFG).

**Results:**

We tested the hypothesis that self-weighing frequency and the incidence of T2D are associated in 2607 people with IFG (1240 in the intervention arm; 1367 in the self-directed control arm). Both arms received a weighing scale with storage function. Healthcare providers offered a one-year goal-focused lifestyle intervention via phone. Participants were divided into 4 categories based on self-weighing frequency (No data sent [reference group], low: < 2 times/week, middle: 3–4 times/week, and high: 5–7 times/week). The adjusted hazard ratio (AHR) and 95% confidence interval (CI) were calculated. In the intervention arm, middle- and high-frequency self-weighing were associated with a decreased incidence of T2D relative to the reference group (AHR = 0.56, 95% CI [0.32, 0.98] and AHR = 0.43, 95% CI [0.25, 0.74], respectively). In the control arm, high-frequency self-weighing was also associated with a decreased incidence of T2D relative to the reference group (AHR = 0.54, 95% CI [0.35, 0.83]).

*Trial registration* This trial has been registered with the University Hospital Medical Information Network (UMIN000000662).

## Introduction

The Japan Diabetes Outcome Trial-1 (J-DOIT1) is a large-scale lifestyle intervention for people with prediabetes [1]. Participants in the J-DOIT1 received a weight scale and were encouraged by healthcare providers to weigh their bodies and make healthy lifestyle changes. Little is known about the relationship between frequent self-weighing and the incidence of type 2 diabetes (T2D). The aim of this study was to examine the association between self-weighing frequency and the incidence of T2D in high-risk individuals using a post hoc analysis of the J-DOIT1 data.

## Main text

### Study design and participants

This study is a post hoc analysis of the cluster-randomized controlled trial J-DOIT1. A total of 43 health care divisions were enrolled across the country (Hokkaido, Tohoku, Kanto, Chubu, Kansai, Chugoku, Shikoku, and Kyushu). Forty-three health care divisions (clusters) were randomly assigned to an intervention arm (22 groups) or a control arm (21 clusters). Detailed information on the design and intervention has already been published elsewhere [2]. The inclusion criteria were impaired fasting glucose (IFG) and an age of 20–65 years. The exclusion criteria included diabetes, anti-diabetic medication use, HbA1c ≥ 6.5%, and contraindications to exercise but not a history of gestational diabetes. This study was conducted in workplaces and communities. The study participants were recruited at health checkups. Healthcare providers, such as public health nurses and dieticians, provided the intervention. The lifestyle support center managed the recruitment and enrollment of the study participants. The lifestyle support center sent a program kit by mail to the eligible subjects in each cluster, inviting them to participate in the study. Participants in both arms received a pedometer (HJ-710 IT; Omron Healthcare Co., Ltd., Japan) and a weight scale (HBF-354 IT-2; Omron Healthcare Co., Ltd., Japan). The participants were encouraged to measure their body weight every day and send the accumulated data to the lifestyle support center via a transmitter (DC-100; JMS Co., Ltd., Japan).

### Intervention arm

Healthcare providers offered a 1-year lifestyle intervention via phone. The target goals were (1) exercise habits (≥ 10,000 steps/day), (2) weight control (a 5% reduction in body weight in obesity), (3) vegetable intake (≥ 350 g/day), and 4) moderate alcohol drinking (≤ 23 g of alcohol/day). The healthcare provider talked with participants about the benefits and barriers of health behavior changes. They made strategies to solve the problems together. Feedback by graph (body weight and footsteps) was provided monthly. The healthcare providers encouraged participants based on the daily step and weight change data. The healthcare providers discussed the benefits and barriers with participants. Self-weighing promotes self-regulation and awareness of one’s weight trend and weight-related behaviors. The benefits of daily self-weighing include immediate feedback on overeating and overdrinking, awareness of weight trends such as weight gain on the weekend, analysis of self-regulatory behavior, motivation for weight management, and recognition of relapse. The barriers include psychological burden, difficulties interpreting weight changes due to unaccountable fluctuations, problems with remembering previous readings, and unrealistic weight loss expectations. The healthcare providers emphasized that being aware of one’s weight is a key factor in successful weight loss. The intervention arm received five to six phone calls (mean 5.6 ± 3.2 calls) per year, with the length of each call being between 15 and 30 min. The first session was an “introduction and welcome call”, and subsequent sessions were “support calls”. During the support call, evaluation of goal achievement and design of a correction plan were performed. During and after the 1-year intervention, the participants in both arms were also encouraged to weigh themselves daily via newsletters provided throughout the intervention.

### Control arm

The goals for lifestyle change were brought up by the healthcare providers or physician at the health care division to each participant in the control arm and based on the following four points: (1) exercise habits, (2) weight control, (3) vegetable intake, and 4) moderate alcohol drinking. After setting the goals, the participants tried to achieve the behavior change goals on their own using a weight scale and pedometer. They received feedback by graph (body weight and footsteps) monthly. Participants in the control arm periodically also received newsletters on diabetes and diabetes prevention but did not receive telephone-delivered interventions. Participants were encouraged, through newsletters, to undergo annual health check-ups. The newsletters included information on the benefits of health behavior changes but not the barriers. The participants were encouraged periodically by newsletters. The control arm was used a self-help device and received newsletters with little professional help.

### Outcome measures

Diabetes was defined as an FPG ≥ 7.0 mmol/L (126 mg/d) or the use of diabetes medications. Body weight, vegetable intake, and alcohol consumption were measured after the one-year intervention and at the end of the trial. Vegetable intake and alcohol consumption were measured using a self-administered questionnaire. Daily steps during the one-year intervention were also measured. Exercise habits were defined as achieving 10,000 steps or more.

### Statistical analysis

The participants were divided into 4 categories based on the frequency of self-weighing (Not data sent [reference group], low: < 2 times/week, middle: 3–4 times/week, and high: 5–7 times/week).

Cox regression analysis was applied to calculate the adjusted HR (AHR) and 95% confidence interval (CI) relative to the reference category. We adjusted for age, sex, and BMI and took into account the clustering effect. The trend test was performed using the Cochran–Armitage test or Jonckheere-Terpstra test. The data were analyzed using Stata/IC ver. 13.1 software and Statistical Package for Social Science software ver. 24.0 (IBM SPSS Inc, Chicago, Illinois, USA).

## Results

There was no difference in age or FPG between categories of self-weighing. In the intervention arm, the % of males tended to increase with self-weighing frequency (Table 1). The prevalence of lean, normal weight, and obese individuals in the intervention arm were 1.9, 59.0, and 39.1%, respectively. The prevalence of lean, normal weight, and obese individuals in the control arm was 2.1, 60.4, and 37.5%, respectively. There was no difference in BMI or BMI categories between the arms (Additional file 1: Table S1). Significant correlations were noted between self-weighing frequency and body weight changes and exercise habits after the intervention and enduring the follow-up period in both arms (Table 2). There was a significant trend between self-weighing frequency and vegetable intake after the intervention and during the follow-up period in the intervention arm, although there was no difference after the intervention in the control arm. The association between self-weighing frequency and the restriction of alcohol drinking was not significant. There was a significant trend between self-weighing frequency and the phone call participation rate in the intervention arm.

**Table 1:** Baseline characteristics of the participants according to categories of self-weighing frequency

Variables	Reference	Low frequency (< 2 times/week)	Middle frequency (2–4 times/week)	High frequency (5–7 times/week)	*P* value
Number (%)					
Intervention arm	289 (23.3%)	285 (23.0%)	281 (22.7%)	385 (31.0%)	–
Control arm	257 (18.8%)	412 (30.1%)	340 (24.9%)	358 (26.2%)	–
Age, years					
Intervention arm	49.9 (8.4)	46.8 (7.8)	48.3 (7.3)	50.1 (7.4)	0.263
Control arm	49.7 (7.5)	47.7 (8.1)	48.4 (7.0)	50.1 (7.1)	0.112
Male, %					
Intervention arm	67.1%	86.3%	89.0%	86.5%	< 0.001
Control arm	77.8%	86.4%	85.0%	85.2%	0.063
BMI, kg/m^2^					
Intervention arm	24.7 ± 3.6	24.4 ± 3.2	24.5 ± 3.0	24.1 ± 2.9	0.092
Control arm	24.7 ± 3.3	24.3 ± 3.0	24.2 ± 3.3	23.9 ± 2.8	0.002
Fasting plasma glucose, mmol/L					
Intervention arm	5.92 ± 0.34	5.92 ± 0.35	5.89 ± 0.33	5.90 ± 0.31	0.988
Control arm	5.90 ± 0.31	5.90 ± 0.33	5.91 ± 0.33	5.89 ± 0.30	0.568
The phone call participation rate, %					
Intervention arm	61.6%	86.6%	92.7%	97.5%	< 0.001
Control arm	–	–	–	–	–

Values are number (%) or the mean (standard deviation). P values using the Cochran–Armitage test or Jonckheere-Terpstra test

**Table 2:** Frequency categories of self-weighing, body weight changes, and healthy lifestyle behavior

Variables	Reference	Low frequency (< 2 times/week)	Middle frequency (2–4 times/week)	High frequency (5–7 times/week)	P-value
Body weight changes, kg					
After 1-year intervention					
Intervention arm	− 0.2 ± 2.4	− 0.6 ± 3.0	− 1.7 ± 3.0	− 2.6 ± 3.7	< 0.001
Control arm	− 0.3 ± 2.9	− 0.3 ± 2.9	− 1.1 ± 3.1	− 1.6 ± 3.6	< 0.001
End of trial					
Intervention arm	0.0 ± 2.3	− 0.4 ± 3.1	− 1.4 ± 3.5	− 2.2 ± 3.8	0.025
Control arm	− 0.3 ± 3.2	0.0 ± 3.5	− 1.0 ± 3 .4	− 1.1 ± 3.8	0.002
Exercise habits, %					
After 1-year intervention					
Intervention arm	51.2%	57.9%	64.4%	76.1%	< 0.001
Control arm	56.4%	59.2%	60.3%	71.2%	< 0.001
End of trial					
Intervention arm	51.9%	55.4%	63.3%	69.4%	0.020
Control arm	54.1%	58.7%	58.5%	64.0%	< 0.001
Vegetable intake (≥ 5 dishes), %					
After 1-year intervention					
Intervention arm	0.7%	0.7%	3.2%	8.1%	< 0.001
Control arm	0.4%	0.7%	0.0%	0.6%	0.846
End of trial					
Intervention arm	7.6%	8.8%	11.7%	19%	< 0.001
Control arm	3.9%	3.6%	6.2%	10.1%	< 0.001
Moderate alcohol consumption (≤ 23 g of ethanol), %					
After 1-year intervention					
Intervention arm	45.0%	44.6%	45.2%	44.9%	0.972
Control arm	45.1%	39.1%	41.2%	43.6%	0.956
End of trial					
Intervention arm	52.9%	47.7%	47.7%	47.8%	0.236
Control arm	46.7%	44.9%	43.5%	47.5%	0.845

Data are means (standard deviation) or percent (%).The mean follow-up period after the intervention was 3.1 years. Exercise habits were defined as achieving 10,000 steps and more. P values using the Cochran–Armitage test or Jonckheere-Terpstra test

A total of 247 subjects developed diabetes during the follow-up period. The mean follow-up period after the intervention was 3.1 years. In the intervention arm, middle- and high-frequency self-weighing were associated with a reduced incidence of T2D relative to the reference group (AHR = 0.56, 95% CI [0.32, 0.98] and AHR = 0.43, 95% CI [0.25, 0.74], respectively). In the control arm, high-frequency self-weighing was also associated with a decreased incidence of T2D relative to the reference group (AHR = 0.54, 95% CI [0.35, 0.83]) (Table 3). Relative to low-frequency self-weighing, high-frequency self-weighing was associated with a decreased incidence of T2D (AHR = 0.65, 95% CI [0.50, 0.86]) in the intervention arm, although middle frequency weighing was not associated with the incidence of T2D (AHR = 0.56, 95% CI [0.29, 1.07]). In the control arm, middle- and high-frequency self-weighing were not associated with a decreased incidence of T2D relative to low-frequency self-weighing (AHR = 0.74, 95% CI [0.41, 1.35] and AHR = 0.80, 95% CI [0.62, 1.04], respectively).

**Table 3:** Frequency categories of self-weighing and the incidence of diabetes

Variables	Reference	Low frequency (< 2 times/week)	Middle frequency (2–4 times/week)	High frequency (5–7 times/week)
Diabetes Incidence, per 100 person-years				
Intervention arm	4.0	2.9	1.6	1.3
Control arm	2.8	2.6	2.0	1.7
Adjusted hazard ratio (95% CI)				
Intervention arm	1.00	0.81 (0.49–1.33)	0.56 (0.32–0.98)	0.43 (0.25–0.74)
Control arm	1.00	0.93 (0.58–1.49)	0.75 (0.47–1.19)	0.54 (0.35–0.83)

Cox regression analysis was used to calculate the HR and 95% CI. We adjusted for age, sex, and BMI and took into account the clustering effect

## Discussion

To our knowledge, this is the first study to report an association between the frequency of self-weighing and the incidence of T2D using objective data from a large-scale randomized controlled trial. Interestingly, this association was observed not only in the intervention arm but also in the control arm.

As shown in Table 2, there was an association between self-weighing frequency and weight reduction relative to baseline. In the most frequent group (i.e., the high-frequency self-weighing group: 5–7 times/week), the decrease in body weight after the 1-year intervention was − 2.6 ± 3.7 kg, which was maintained even at the end of the trial (− 2.2 ± 3.8 kg). There were also positive associations between self-weighing frequency and the percent of subjects who achieved the goals set regarding exercise habits and vegetable intake. Furthermore, we found that the incidence of diabetes in the middle- and high-frequency self-weighing groups was significantly less than that in the low-frequency or reference group, as shown in Table 3. Thus, using the data obtained in the J-DOIT1, we confirmed previously reported findings that frequent self-weighing is effective for weight reduction. Moreover, the results suggested that frequent self-weighing could lead to a decrease in the development of diabetes.

Importantly, those favorable changes were observed even in the control arm. The participants were self-directed and used self-help devices. They periodically received a newsletter from the lifestyle support center, which contained health-related information, but they did not receive telephone-delivered lifestyle coaching by healthcare professionals. They were provided with the same type of weight scales. They were encouraged to measure their body weight every day and send the accumulated data to the lifestyle support center monthly. However, unlike those in the intervention arm, they did not receive feedback. They received only graphic records of daily body weight from the lifestyle support center without comments. Nevertheless, 358 (26.2%) subjects measured their body weight 5–7 times/week during the 1-year period. They showed a small but significant level of body weight reduction (− 1.6 ± 3.6 kg after 1 year and − 1.1 ± 3.8 kg at the end of the trial). There were increases in the number of subjects who achieved the goals set for exercise habits and vegetable intake. The incidence of diabetes was significantly decreased in the high-frequency self-weighing group to a level almost comparable to that observed in the intervention arm. The results suggest that prevention of T2D may be possible in a real-world setting using self-help health devices with little professional help.

Monitoring body weight might allow people to notice how specific patterns or situations of physical or eating behavior are associated with body weight. Additionally, they give positive reinforcement when changes in behavior correspond to avoidance of weight gain or weight loss [3]. Adding a recommendation for daily self-weighing to lifestyle modification programs could reduce the incidence of T2D in a real-world setting. Self-weighing and immediate feedback is a low-cost and effective tool for promoting weight loss [4]. An increased self-weighing frequency was associated with greater weight loss among 18- to 25-year-olds during a brief lifestyle intervention [5]. In this study, an increased self-weighing frequency was associated with weight changes. In a 12-month community Diabetes Prevention Program (DPP) lifestyle intervention, self-weighing behavior was associated with weight loss [6]. In a weight loss trial, self-weighing behavior led to significant weight loss [7]. Therefore, frequent self-weighing might reduce the incidence of T2D.

An increased self-weighing frequency was associated with exercise habits and vegetable intake. Several studies have shown that moderate to intense levels of exercise habits are effective for reducing the risk of T2D [8]. A meta-analysis involving overweight and obese adults with T2D showed that pedometer use led to a significantly decreased BMI (-0.15 kg/m^2^) and reduced weight (-0.65 kg) [9]. Frequent self-weighing combined with the use of a pedometer might promote weight loss in prediabetic subjects.

In DPP, a low-fat diet and high-fiber intake with overall calorie reduction led to significant weight loss, which may reduce the incidence of T2D in high-risk people [10]. Dietary fiber intake is related to increased satiety and fullness [11]. These comprehensive factors might explain why the intervention reduced the incidence of T2D.

## Limitations

The strengths of this study include the use of objective data from self-weighing, a long follow-up duration, and a large sample size. However, there are several limitations. The reference category may have included participants who frequently performed self-weighing, but this could not be confirmed because they did not send data. The phone call participation rate in the reference category was lower. Careful attention should be paid when interpreting these results.

## Supplementary information

**Additional file 1: Table S1.** Baseline characteristics of the participants according to categories of self-weighing frequency.

## Data Availability

The datasets used and analyzed in the current study are available from the corresponding author on reasonable request.

## Abbreviations

IFG: Impaired fasting glucose
J-DOIT1: The Japan Diabetes Outcome Trial-1
T2D: Type 2 diabetes
