# Supplementary material for: Self-weighing frequency and the incidence of type 2 diabetes: post hoc analysis of a cluster-randomized controlled trial
Source: BMC Res Notes. 2020 Aug 8;13:375. doi: 10.1186/s13104-020-05215-x (PMC7414687; doi:10.1186/s13104-020-05215-x)
Supplement: Supplementary file 1 — Additional file 1: Table S1. Baseline characteristics of the participants according to categories of self-weighing frequency. [file 13104_2020_5215_MOESM1_ESM.docx]

Additional file 1: Table S1. Baseline characteristics of the participants according to categories of self-weighing frequency

| BMI categories | Reference | Low frequency | Middle frequency | High frequency |
| --- | --- | --- | --- | --- |
|  |  | (<2 times/week) | (2-4 times/week) | (5-7 times/week) |
| Intervention arm, %  Lean (<18.5)  Normal weight (18.5-24.9  Obesity (>25) | 8  158  123 | 5  165  115 | 3  165  113 | 8  243  134 |
| Control arm, %  Lean (<18.5)  Normal weight (18.5-24.9  Obesity (>25) | 6  140  111 | 8  250  154 | 9  194  137 | 6  241  111 |
| P value | 0.943 | 0.742 | 0.396 | 0.488 |

Values are number (%).
